# Coupling between 2-pyridyl­selenyl chloride and phenyl­seleno­cyanate: synthesis, crystal structure and non-covalent inter­actions

**DOI:** 10.1107/S2056989024008831

**Published:** 2024-09-17

**Authors:** Ayalew W. Temesgen, Alexander G. Tskhovrebov, Alexey A. Artemjev, Alexey S. Kubasov, Alexander S. Novikov, Alexander V. Borisov, Anatoly A. Kirichuk, Andreii S. Kritchenkov, Tuan Anh Le

**Affiliations:** a Department of Chemistry, College of Natural and Computational Science, University of Gondar, Gondar 196, Ethiopia; b People’s Friendship University of Russia 6 Miklukho-Maklaya Street Moscow 117198 Russian Federation; c Kurnakov Institute of General and Inorganic Chemistry, Russian Academy of Sciences, Leninsky Prosp. 31, 119071, Moscow, Russian Federation; d Institute of Chemistry, Saint Petersburg State University, Universitetskaya, Nab., 7/9, 199034 Saint Petersburg, Russian Federation; e R.E. Alekseev Nizhny Novgorod State Technical University Minin St 24 Nizhny Novgorod Russian Federation; f University of Science Vietnam National University, Hanoi, 334 Nguyen Trai Thanh Xuan Hanoi 100000 Vietnam; Universidad de Los Andes, Venezuela

**Keywords:** crystal structure, non-covalent inter­actions, chalcogen heterocycles, chalcogen bonding

## Abstract

A new pyridine-fused seleno­diazo­lium salt, 3-(phenyl­selan­yl)[1,2,4]selena­diazolo[4,5-*a*]pyridin-4-ylium chloride di­chloro­methane 0.352-solvate, C_12_H_9_N_2_Se_2_^+^·Cl^−^·0.352CH_2_Cl_2_, was obtained from the reaction between 2-pyridyl­selenenyl chloride and phenyl­seleno­cyanate. Single-crystal structural analysis revealed the presence of C—H⋯N, C—H⋯Cl^−^, C—H⋯Se hydrogen bonds as well as chalcogen–chalcogen (Se⋯Se) and chalcogen–halogen (Se⋯Cl^−^) inter­actions. Non-covalent inter­actions were explored by DFT calculations followed by topological analysis of the electron density distribution (QTAIM analysis). The structure consists of pairs of seleno­diazo­lium moieties arranged in a head-to-tail fashion surrounding disordered di­chloro­methane mol­ecules. The assemblies are connected by C—H⋯Cl^−^ and C—H⋯N hydrogen bonds, forming layers, which stack along the *c*-axis direction connected by bifurcated Se⋯Cl^−^⋯H—C inter­actions.

## Abstract 2

The structure of a new seleno­diazo­lium salt derived from the reaction between 2-pyridyl­selenyl chloride and phenyl­seleno­cyanate is described. Halogen–hydrogen, chalcogen–chalcogen, chalcogen–hydrogen and chalcogen–halogen inter­actions are present in the structure.

## 1. Chemical context

Recently, a novel cyclo­addition reaction between nitriles and 2-pyridyl­selenyl reagents was described (Khrustalev *et al.*, 2021 ▸). What makes this finding particularly notable is that the reaction takes place under mild conditions, displaying a high degree of chemoselectivity (Grudova *et al.*, 2022 ▸; Artemjev *et al.*, 2023 ▸). As a result, pyridinium-fused seleno­diazo­lium salts are formed with excellent yields.

As part of our ongoing project to investigate the reactivity of bifunctional 2-pyridyl­selenyl reagents (Grudova *et al.*, 2022 ▸; Artemjev *et al.*, 2022 ▸, 2023 ▸; Sapronov *et al.*, 2023 ▸) we have recently expanded our research to explore the chemistry of addition to the C≡N triple bond involving a different category of nitrile substrates known as cyanamides or push–pull nitriles. Push–pull structures are characterized by high polarization and consist of an electron-withdrawing substituent or electronegative atom on one side of the multiple bond and an electron-donating group on the opposite side (Le Questel *et al.*, 2000 ▸; Gushchin *et al.*, 2009 ▸; Kritchenkov *et al.*, 2011 ▸).

Here we show that 2-pyridyl­selenyl chloride reacts efficiently with phenyl­seleno­cyanate furnishing a cationic pyridinium-fused 1,2,4-seleno­diazole in high yield. This finding is another illustration of the remarkable propensity of bifunctional 2-pyridyl­selenyl reagents to engage in dipolar cyclo­addition with the CN triple bond, displaying a high degree of chemoselectivity. The title compound was synthesized in high yield in CH_2_Cl_2_ according to the scheme.
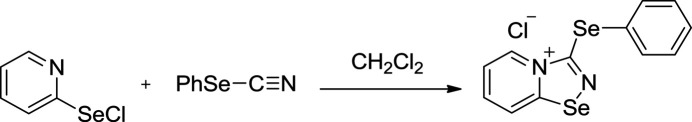


## 2. Structural commentary

**Figure 1:**
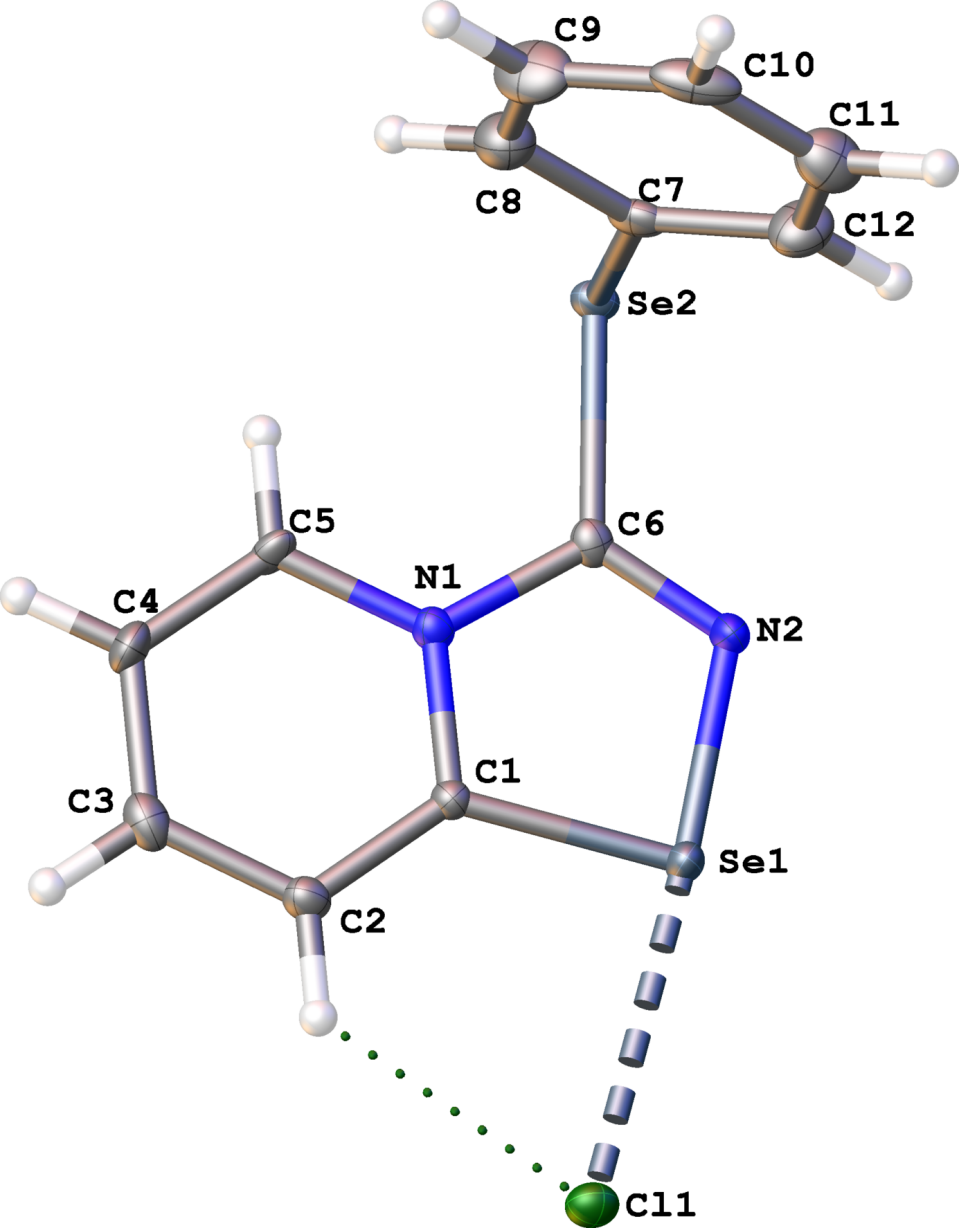
Mol­ecular structure of the title compound. Displacement ellipsoids are drawn at the 50% probability level.

Crystals suitable for X-ray analysis were obtained directly from the reaction mixture. The compound crystallized as colorless blocks in space group *P*2_1_/*c*. The asymmetric unit (Fig. 1 ▸) contains one cation, one Cl^−^ anion and a disordered CH_2_Cl_2_ mol­ecule. The 1,2,4-seleno­diazole fragment is almost planar (r.m.s.d. = 0.017 Å) and makes an angle of 81.64 (16)° with the phenyl­selenyl ring. The Se1—N2 and Se1—C1 bond lengths are 1.863 (4) and 1.877 (4) Å, respectively, and the Se1⋯Cl1 distance is 2.9325 (17). These bond distances are similar to those reported in previous work on 1,2,4-seleno­diazo­les (Grudova *et al.*, 2022 ▸; Artemjev *et al.*, 2022 ▸, 2023 ▸; Sapronov *et al.*, 2022 ▸, 2023 ▸). The Se2—C6 and Se2—C7 bond lengths are typical for Se—C_ar_ bonds [1.926 (5) Å and 1.946 (5) Å, respectively]. The C7—Se2—C6—N1 and C6—Se2—C7—C12 torsion angles are 93.4 (5) and 76.9 (4)°, respectively.

## 3. Supra­molecular features

**Figure 2:**
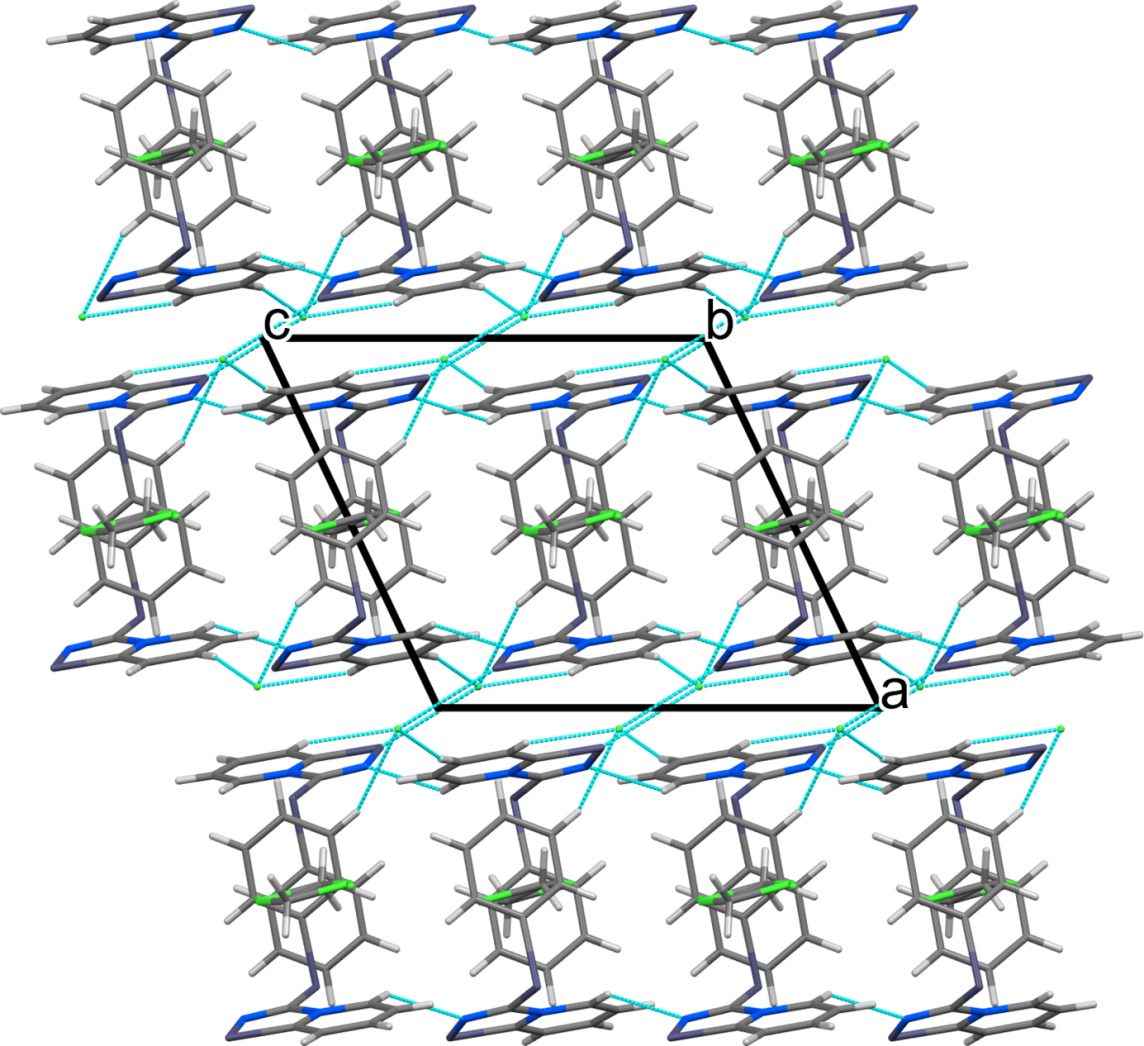
View along the *b* axis of the crystal packing of the title compound.

The crystal packing is shown in Fig. 2 ▸, viewed down the *b* axis. In the crystal, pairs of seleno­diazo­lium moieties are arranged in a head-to-tail fashion surrounding disordered di­chloro­methane mol­ecules. C—H⋯Cl^−^ and C—H⋯N hydrogen bonds (Table 1 ▸) connect these units to form layers parallel to the *ac* plane. In addition, π–π stacking inter­actions between the phenyl rings of two neighboring mol­ecules occur. The layers are inter­connected via bifurcated Se⋯Cl^−^⋯H—C inter­actions and stack along the *c*-axis.

**Figure 3:**
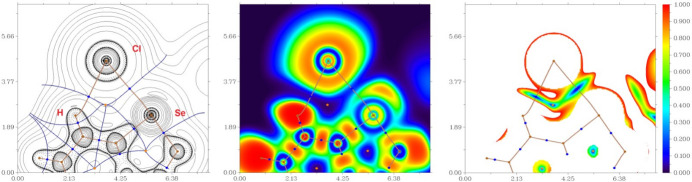
Contour line diagram of the Laplacian of the electron-density distribution *Ñ*^2^*r*(**r**), bond paths, and selected zero-flux surfaces (left panel), visualization of the electron localization function (ELF, center panel) and reduced density gradient (RDG, right panel) analyses for bifurcated chalcogen-hydrogen bonding Se⋯Cl^−^⋯H—C (contacts Se1⋯Cl1^−^ 2.9325 (17) Å and C2—H2⋯Cl^−^ 2.60 Å) in the crystal structure. Bond critical points (3, −1) are shown in blue, nuclear critical points (3, −3) in pale brown, ring critical points (3, +1) in orange, bond paths are shown as pale brown lines, length units in Å, and the color scale for the ELF and RDG maps is presented in a.u.

To further understand the nature of the inter­actions and to qu­antify the strength of the bifurcated chalcogen–halogen–hydrogen contacts, Se⋯Cl^−^⋯H—C, and the inter­actions involving the Se atom (Se⋯Se and Se⋯Cl^−^) in the crystal structure, DFT calculations followed by a topological analysis of the electron-density distribution (QTAIM analysis) were carried out at the ωB97XD/6-311++G** level of theory for the model structure (see Computational details and Table S1 in the supporting information). The results of the QTAIM analysis are summarized in Table S1. The contour line diagrams of the Laplacian of the electron density distribution *Ñ*^2^*r*(**r**), bond paths, and selected zero-flux surfaces, visualization of electron localization function (ELF) and reduced density gradient (RDG) analyses for bifurcated Se⋯Cl^−^⋯H—C, Se⋯Se and Se⋯Cl^−^ inter­actions in the crystal structure are shown in Figs. 3 ▸ and 4 ▸, respectively.

The QTAIM analysis of the model structure demonstrates the presence of bond critical points (3, −1) for short contacts Se⋯Cl^−^, C–H⋯Cl^−^ and Se⋯Se in the crystal structure (Table S1 and Figs. 3 ▸ and 4 ▸) (Bondi *et al.*, 1966 ▸). The low magnitude of the electron density, the positive values of the Laplacian of the electron density and zero or very close to zero values of the energy density in these bond critical points (3, −1) and estimated strength for appropriate short contacts are typical for weak purely non-covalent [–*G*(**r**)/*V*(**r**) > 1; Espinosa *et al.*, 2002 ▸] inter­actions. The Laplacian of the electron density is typically decomposed into the sum of contributions along the three principal axes of maximal variation. The three eigenvalues of the Hessian matrix (λ_1_, λ_2_ and λ_3_) and the sign of λ_2_ can be utilized to distinguish bonding (attractive, λ_2_ < 0) weak inter­actions from non-bonding ones (repulsive, λ_2_ > 0) (Johnson *et al.*, 2010 ▸; Contreras-García *et al.*, 2011 ▸). Thus, the discussed short contacts Se⋯Cl^−^, C–H⋯Cl^−^ and Se⋯Se in the structure are attractive.

## 4. Database survey

A search in the Cambridge Structural Database (CSD, Version 5.43, update of Sep. 2022; Groom *et al.*, 2016 ▸) showed only 16 hits for 1,2,4-seleno­diazo­lium salts, which differ not only in the type of nitrile fragment (Me: EWEPUU, Khrustalev *et al.*, 2021 ▸; Ph: NAQDES, Buslov *et al.*, 2021 ▸; BrC_6_H_4_: EWEQEF, Khrustalev *et al.*, 2021 ▸), but also in the anion (CF_3_COO–: YEJXEU; AuCl_4_–: YEJXUK; and ReO_4_^−^: YEJYAR, Artemjev *et al.*, 2022 ▸).

## 5. Synthesis and crystallization

**General remarks**. All manipulations were carried out in air. All the reagents used in this study were obtained from commercial sources (Aldrich, TCI-Europe, Strem, ABCR). Commercially available solvents were purified by conventional methods and distilled right before they were used. NMR spectra were recorded on a Bruker Advance Neo (^1^H: 700 MHz); chemical shifts (*δ*) are given in ppm, coupling constants (*J*) in Hz. 2-Pyridyl­selenyl chloride was synthesized by our method (Artemjev *et al.*, 2022 ▸; Artemjev *et al.*, 2023 ▸).

A solution of phenyl­seleno­cyanate (0.16 mmol, 20 µL) in CH_2_Cl_2_ (1 mL) was added to a suspension of 2-pyridyl­selenyl chloride (0.13 mmol, 25.3 mg) in CH_2_Cl_2_ (2 mL) and the mixture was kept at room temperature for 6 h without stirring. The formed colorless precipitate was centrifuged, washed with CH_2_Cl_2_ (1 mL), Et_2_O (3 × 1 mL) and dried under vacuum. Yield 34.5 mg (70%). ^1^H NMR (700 MHz, D_2_O) *δ* 9.53 (*d*, *J* = 6.8 Hz, 1H, H5), 8.82 (*d*, *J* = 8.6 Hz, 1H, H8), 8.42 (*t*, *J* = 7.9 Hz, 1H, H7), 8.05 (*t*, *J* = 7.0 Hz, 1H, H6), 7.82 (*d*, *J* = 7.6 Hz, 2H, H2′), 7.55 (*t*, *J* = 7.5 Hz, 1H, H4′), 7.49 (*t*, *J* = 7.7 Hz, 2H, H3′). ^13^C NMR (176 MHz, D_2_O) *δ* 168.1 (C3), 148.8 (C9), 139.9 (C5), 137.4 (C8), 135.7 (C2′), 130.5 (C4′), 130.3 (C3′), 126.0 (C7), 123.8 (C1′), 123.2 (C6). Crystals suitable for X-ray analysis were obtained directly from the reaction mixture.

The single-point calculations based on the experimental X-ray structure were carried out at the DFT level of theory using the dispersion-corrected hybrid functional ωB97XD (Chai *et al.*, 2008 ▸) with the *Gaussian-09* (Frisch *et al.*, 2010 ▸) program package. The 6-311++G** basis sets were used for all atoms. The topological analysis of the electron density distribution was performed using the *Multiwfn* program (version 3.7; Lu *et al.*, 2012 ▸). The Cartesian atomic coordinates for the model structure are presented in Table S1 of the supporting information.

## 6. Refinement

Crystal data, data collection and structure refinement details are summarized in Table 2 ▸. H atoms were included in calculated positions (C—H = 0.95–1.00 Å) and refined as riding with *U*_iso_(H) = 1.2*U*_eq_(C). The di­chloro­methane mol­ecule is disordered around a center of symmetry and refined to a total occupancy of 70%. Residual electron density of 1.5 e Å^−3^ remained at the center of symmetry. Attempts to rationalize it did not produce a plausible model nor an improved refinement.

## Supplementary Material

Crystal structure: contains datablock(s) I. DOI: 10.1107/S2056989024008831/jw2003sup1.cif

Structure factors: contains datablock(s) I. DOI: 10.1107/S2056989024008831/jw2003Isup2.hkl

QTAIM analysis. DOI: 10.1107/S2056989024008831/jw2003sup4.docx

CCDC reference: 2382988

Additional supporting information:  crystallographic information; 3D view; checkCIF report

## Figures and Tables

**Figure 4:**
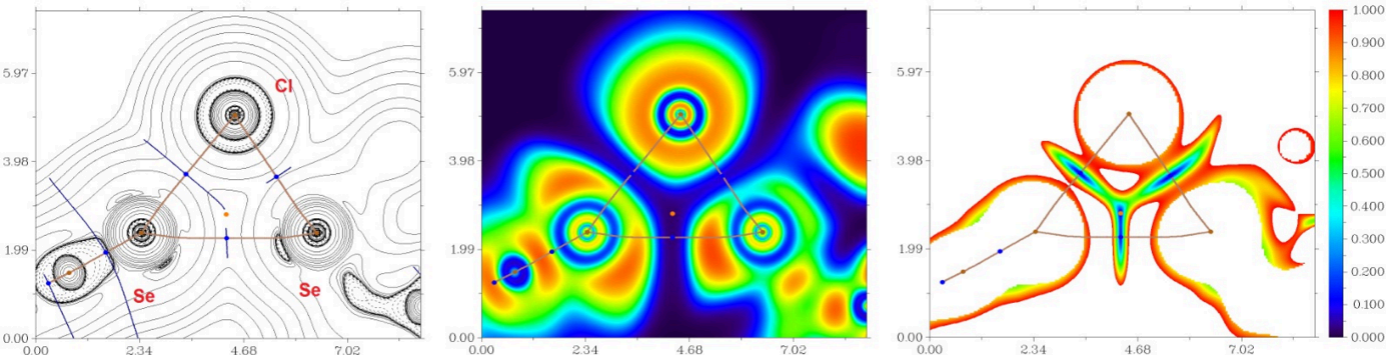
Contour line diagram of the Laplacian of the electron-density distribution *Ñ*^2^*r*(**r**), bond paths, and selected zero-flux surfaces (left panel), visualization of the electron localization function (ELF, center panel) and reduced density gradient (RDG, right panel) analyses for chalcogen bonding Se2⋯Se1^i^ [3.9426 (18) Å; symmetry code: (i) *x*, 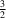 − *y*, 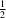 + *z*], Se2⋯Cl1^–ii^ [3.229 (2) Å; symmetry code: (ii) −*x*, 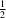 + *y*, 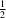 − *z*] and Se1⋯Cl1^–iii^ [3.3805 (19) Å; symmetry code: (iii) −*x*, 1 − *y*, −*z*] in the crystal structure. Bond critical points (3, −1) are shown in blue, nuclear critical points (3, −3) in pale brown, ring critical points (3, +1) in orange, bond paths are shown as pale brown lines, length units in Å, and the color scale for the ELF and RDG maps is presented in a.u.

**Table 1:** Hydrogen-bond geometry (Å, °)

*D*—H⋯*A*	*D*—H	H⋯*A*	*D*⋯*A*	*D*—H⋯*A*
C2—H2⋯Cl1	0.95	2.60	3.297 (5)	131
C3—H3⋯Cl1^i^	0.95	2.60	3.526 (5)	167
C5—H5⋯Se2	0.95	2.82	3.242 (5)	108
C5—H5⋯N2^ii^	0.95	2.45	3.179 (6)	134

Symmetry codes: (i) 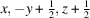; (ii) 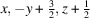.

**Table 2:** Experimental details

Crystal data	
Chemical formula	C_12_H_9_N_2_Se_2_^+^·Cl^−^·0.352CH_2_Cl_2_
*M*_r_	404.50
Crystal system, space group	Monoclinic, *P*2_1_/*c*
Temperature (K)	100
*a*, *b*, *c* (Å)	11.087 (3), 11.758 (5), 11.991 (3)
β (°)	115.337 (6)
*V* (Å^3^)	1412.8 (8)
*Z*	4
Radiation type	Mo *K*α
μ (mm^−1^)	5.54
Crystal size (mm)	0.20 × 0.10 × 0.08

Data collection	
Diffractometer	Bruker D8 Venture
Absorption correction	Multi-scan (*SADABS*; Krause *et al.*, 2015 ▸)
*T*_min_, *T*_max_	0.466, 0.746
No. of measured, independent and observed [*I* > 2σ(*I*)] reflections	7927, 3394, 2649
*R*_int_	0.038
(sin θ/λ)_max_ (Å^−1^)	0.661

Refinement	
*R*[*F*^2^ > 2σ(*F*^2^)], *wR*(*F*^2^), *S*	0.043, 0.088, 1.06
No. of reflections	3394
No. of parameters	182
No. of restraints	20
H-atom treatment	H-atom parameters constrained
Δρ_max_, Δρ_min_ (e Å^−3^)	1.50, −0.92

Computer programs: *APEX2* and *SAINT* (Bruker, 2019 ▸), *SHELXT2014/5* (Sheldrick, 2015*a* ▸), *SHELXL2019/2* (Sheldrick, 2015*b* ▸) and *OLEX2* (Dolomanov *et al.*, 2009 ▸).

## supplementary crystallographic information

### 3-(Phenylselanyl)[1,2,4]selenadiazolo[4,5-a]pyridin-4-ylium chloride dichloromethane Crystal data

**Table:**

C_12_H_9_N_2_Se_2_^+^·Cl^−^·0.352CH_2_Cl_2_	*F*(000) = 779
*M**_r_* = 404.50	*D*_x_ = 1.902 Mg m^−^^3^
Monoclinic, *P*2_1_/*c*	Mo *K*α radiation, λ = 0.71073 Å
*a* = 11.087 (3) Å	Cell parameters from 3887 reflections
*b* = 11.758 (5) Å	θ = 2.6–28.0°
*c* = 11.991 (3) Å	µ = 5.54 mm^−^^1^
β = 115.337 (6)°	*T* = 100 K
*V* = 1412.8 (8) Å^3^	Block, colourless
*Z* = 4	0.20 × 0.10 × 0.08 mm

### 3-(Phenylselanyl)[1,2,4]selenadiazolo[4,5-a]pyridin-4-ylium chloride dichloromethane Data collection

**Table:**

Bruker D8 Venture diffractometer	2649 reflections with *I* > 2σ(*I*)
φ and ω scans	*R*_int_ = 0.038
Absorption correction: multi-scan (SADABS; Krause *et al.*, 2015)	θ_max_ = 28.0°, θ_min_ = 2.0°
*T*_min_ = 0.466, *T*_max_ = 0.746	*h* = −12→14
7927 measured reflections	*k* = −12→15
3394 independent reflections	*l* = −15→15

### 3-(Phenylselanyl)[1,2,4]selenadiazolo[4,5-a]pyridin-4-ylium chloride dichloromethane Refinement

**Table:**

Refinement on *F*^2^	Primary atom site location: dual
Least-squares matrix: full	Secondary atom site location: difference Fourier map
*R*[*F*^2^ > 2σ(*F*^2^)] = 0.043	Hydrogen site location: mixed
*wR*(*F*^2^) = 0.088	H-atom parameters constrained
*S* = 1.06	*w* = 1/[σ^2^(*F*_o_^2^) + (0.0153*P*)^2^ + 6.8557*P*] where *P* = (*F*_o_^2^ + 2*F*_c_^2^)/3
3394 reflections	(Δ/σ)_max_ < 0.001
182 parameters	Δρ_max_ = 1.50 e Å^−^^3^
20 restraints	Δρ_min_ = −0.91 e Å^−^^3^

### 3-(Phenylselanyl)[1,2,4]selenadiazolo[4,5-a]pyridin-4-ylium chloride dichloromethane Special details

**Table:**

Geometry. All esds (except the esd in the dihedral angle between two l.s. planes) are estimated using the full covariance matrix. The cell esds are taken into account individually in the estimation of esds in distances, angles and torsion angles; correlations between esds in cell parameters are only used when they are defined by crystal symmetry. An approximate (isotropic) treatment of cell esds is used for estimating esds involving l.s. planes.

### 3-(Phenylselanyl)[1,2,4]selenadiazolo[4,5-a]pyridin-4-ylium chloride dichloromethane Fractional atomic coordinates and isotropic or equivalent isotropic displacement parameters (Å^2^)

**Table:**

	*x*	*y*	*z*	*U*_iso_*/*U*_eq_	Occ. (<1)
Se1	0.10761 (5)	0.56698 (4)	0.17359 (4)	0.01592 (12)	
Se2	0.24558 (5)	0.87121 (4)	0.42629 (4)	0.01426 (12)	
N1	0.1762 (4)	0.6289 (3)	0.4071 (4)	0.0132 (8)	
N2	0.1584 (4)	0.7155 (3)	0.2272 (4)	0.0162 (8)	
C1	0.1373 (4)	0.5329 (4)	0.3362 (4)	0.0121 (9)	
C2	0.1221 (5)	0.4305 (4)	0.3880 (4)	0.0150 (9)	
H2	0.095583	0.363323	0.339559	0.018*	
C3	0.1465 (5)	0.4290 (4)	0.5109 (5)	0.0188 (10)	
H3	0.138254	0.359887	0.548151	0.023*	
C4	0.1833 (5)	0.5294 (4)	0.5812 (4)	0.0183 (10)	
H4	0.197564	0.528274	0.665125	0.022*	
C5	0.1988 (5)	0.6283 (4)	0.5295 (4)	0.0143 (9)	
H5	0.224770	0.695960	0.577185	0.017*	
C6	0.1880 (5)	0.7279 (4)	0.3425 (4)	0.0134 (9)	
C7	0.4343 (5)	0.8524 (4)	0.4672 (5)	0.0162 (10)	
C8	0.5220 (5)	0.8288 (4)	0.5882 (5)	0.0259 (12)	
H8	0.488948	0.818120	0.648615	0.031*	
C9	0.6579 (6)	0.8207 (5)	0.6214 (5)	0.0319 (13)	
H9	0.717735	0.804495	0.704425	0.038*	
C10	0.7062 (5)	0.8362 (4)	0.5339 (6)	0.0281 (13)	
H10	0.799414	0.831392	0.556943	0.034*	
C11	0.6195 (5)	0.8587 (5)	0.4130 (5)	0.0276 (12)	
H11	0.653058	0.868078	0.352710	0.033*	
C12	0.4828 (5)	0.8677 (5)	0.3788 (5)	0.0238 (11)	
H12	0.423253	0.884189	0.295774	0.029*	
Cl1	0.05719 (12)	0.32474 (9)	0.11352 (11)	0.0163 (2)	
Cl2	0.5173 (17)	0.4244 (7)	0.6117 (12)	0.055 (2)	0.352 (3)
Cl3	0.4753 (18)	0.5382 (9)	0.3886 (13)	0.077 (3)	0.352 (3)
C13	0.5222 (19)	0.5578 (8)	0.5473 (13)	0.058 (4)	0.352 (3)
H13A	0.613356	0.590037	0.587480	0.069*	0.352 (3)
H13B	0.459970	0.611151	0.559777	0.069*	0.352 (3)

### 3-(Phenylselanyl)[1,2,4]selenadiazolo[4,5-a]pyridin-4-ylium chloride dichloromethane Atomic displacement parameters (Å^2^)

**Table:**

	*U*^11^	*U*^22^	*U*^33^	*U*^12^	*U*^13^	*U*^23^
Se1	0.0243 (3)	0.0133 (2)	0.0105 (2)	−0.0043 (2)	0.0078 (2)	−0.00122 (19)
Se2	0.0161 (2)	0.0113 (2)	0.0162 (2)	−0.00220 (19)	0.0076 (2)	−0.00216 (19)
N1	0.016 (2)	0.0113 (17)	0.014 (2)	−0.0002 (16)	0.0076 (17)	−0.0001 (16)
N2	0.023 (2)	0.0122 (18)	0.013 (2)	−0.0050 (17)	0.0073 (18)	0.0000 (16)
C1	0.013 (2)	0.012 (2)	0.012 (2)	−0.0013 (18)	0.0052 (19)	0.0001 (17)
C2	0.017 (2)	0.012 (2)	0.016 (2)	−0.0004 (19)	0.008 (2)	−0.0013 (19)
C3	0.018 (2)	0.018 (2)	0.021 (3)	0.000 (2)	0.009 (2)	0.005 (2)
C4	0.024 (3)	0.021 (2)	0.010 (2)	−0.002 (2)	0.008 (2)	−0.0019 (19)
C5	0.021 (2)	0.013 (2)	0.009 (2)	0.005 (2)	0.0062 (19)	−0.0008 (18)
C6	0.014 (2)	0.013 (2)	0.015 (2)	0.0005 (18)	0.007 (2)	0.0008 (18)
C7	0.015 (2)	0.009 (2)	0.022 (3)	−0.0017 (18)	0.005 (2)	−0.0016 (19)
C8	0.024 (3)	0.027 (3)	0.023 (3)	−0.003 (2)	0.008 (2)	−0.002 (2)
C9	0.019 (3)	0.032 (3)	0.029 (3)	0.001 (2)	−0.005 (2)	0.001 (3)
C10	0.014 (3)	0.016 (2)	0.049 (4)	0.000 (2)	0.008 (3)	−0.007 (2)
C11	0.025 (3)	0.028 (3)	0.034 (3)	−0.001 (2)	0.016 (3)	−0.003 (2)
C12	0.017 (3)	0.029 (3)	0.023 (3)	0.000 (2)	0.006 (2)	−0.002 (2)
Cl1	0.0179 (6)	0.0140 (5)	0.0172 (6)	−0.0004 (5)	0.0078 (5)	−0.0028 (4)
Cl2	0.043 (3)	0.040 (5)	0.088 (4)	−0.012 (4)	0.033 (3)	−0.019 (4)
Cl3	0.047 (4)	0.070 (7)	0.113 (5)	−0.004 (6)	0.035 (4)	−0.004 (6)
C13	0.036 (6)	0.049 (7)	0.096 (7)	−0.013 (7)	0.036 (6)	0.005 (7)

### 3-(Phenylselanyl)[1,2,4]selenadiazolo[4,5-a]pyridin-4-ylium chloride dichloromethane Geometric parameters (Å, º)

**Table:**

Se1—N2	1.863 (4)	C7—C8	1.385 (7)
Se1—C1	1.877 (4)	C7—C12	1.390 (7)
Se2—C6	1.926 (5)	C8—C9	1.388 (7)
Se2—C7	1.946 (5)	C8—H8	0.9500
N1—C1	1.367 (6)	C9—C10	1.379 (8)
N1—C5	1.380 (6)	C9—H9	0.9500
N1—C6	1.435 (6)	C10—C11	1.380 (8)
N2—C6	1.285 (6)	C10—H10	0.9500
C1—C2	1.396 (6)	C11—C12	1.395 (7)
C2—C3	1.380 (6)	C11—H11	0.9500
C2—H2	0.9500	C12—H12	0.9500
C3—C4	1.405 (7)	Cl2—C13	1.7596 (12)
C3—H3	0.9500	Cl3—C13	1.7597 (11)
C4—C5	1.362 (6)	C13—H13A	0.9900
C4—H4	0.9500	C13—H13B	0.9900
C5—H5	0.9500		
			
N2—Se1—C1	87.06 (18)	C8—C7—C12	119.8 (5)
C6—Se2—C7	96.45 (18)	C8—C7—Se2	118.9 (4)
C1—N1—C5	121.5 (4)	C12—C7—Se2	121.2 (4)
C1—N1—C6	114.4 (4)	C7—C8—C9	120.2 (5)
C5—N1—C6	124.2 (4)	C7—C8—H8	119.9
C6—N2—Se1	112.2 (3)	C9—C8—H8	119.9
N1—C1—C2	120.1 (4)	C10—C9—C8	120.0 (5)
N1—C1—Se1	109.7 (3)	C10—C9—H9	120.0
C2—C1—Se1	130.2 (3)	C8—C9—H9	120.0
C3—C2—C1	118.7 (4)	C9—C10—C11	120.1 (5)
C3—C2—H2	120.6	C9—C10—H10	119.9
C1—C2—H2	120.6	C11—C10—H10	119.9
C2—C3—C4	120.2 (4)	C10—C11—C12	120.3 (5)
C2—C3—H3	119.9	C10—C11—H11	119.9
C4—C3—H3	119.9	C12—C11—H11	119.9
C5—C4—C3	120.3 (4)	C7—C12—C11	119.5 (5)
C5—C4—H4	119.8	C7—C12—H12	120.2
C3—C4—H4	119.8	C11—C12—H12	120.2
C4—C5—N1	119.2 (4)	Cl2—C13—Cl3	107.9 (5)
C4—C5—H5	120.4	Cl2—C13—H13A	110.1
N1—C5—H5	120.4	Cl3—C13—H13A	110.1
N2—C6—N1	116.6 (4)	Cl2—C13—H13B	110.1
N2—C6—Se2	122.4 (3)	Cl3—C13—H13B	110.1
N1—C6—Se2	121.0 (3)	H13A—C13—H13B	108.4
			
C1—Se1—N2—C6	−0.4 (4)	Se1—N2—C6—N1	−0.5 (5)
C5—N1—C1—C2	−1.3 (7)	Se1—N2—C6—Se2	179.5 (2)
C6—N1—C1—C2	179.9 (4)	C1—N1—C6—N2	1.5 (6)
C5—N1—C1—Se1	177.1 (3)	C5—N1—C6—N2	−177.3 (4)
C6—N1—C1—Se1	−1.6 (5)	C1—N1—C6—Se2	−178.6 (3)
N2—Se1—C1—N1	1.1 (3)	C5—N1—C6—Se2	2.7 (6)
N2—Se1—C1—C2	179.4 (5)	C12—C7—C8—C9	0.1 (8)
N1—C1—C2—C3	0.4 (7)	Se2—C7—C8—C9	−176.7 (4)
Se1—C1—C2—C3	−177.7 (4)	C7—C8—C9—C10	0.0 (8)
C1—C2—C3—C4	1.1 (7)	C8—C9—C10—C11	−0.6 (8)
C2—C3—C4—C5	−1.7 (7)	C9—C10—C11—C12	1.0 (8)
C3—C4—C5—N1	0.8 (7)	C8—C7—C12—C11	0.3 (8)
C1—N1—C5—C4	0.7 (7)	Se2—C7—C12—C11	177.0 (4)
C6—N1—C5—C4	179.4 (4)	C10—C11—C12—C7	−0.8 (8)

### 3-(Phenylselanyl)[1,2,4]selenadiazolo[4,5-a]pyridin-4-ylium chloride dichloromethane Hydrogen-bond geometry (Å, º)

**Table:**

*D*—H···*A*	*D*—H	H···*A*	*D*···*A*	*D*—H···*A*
C2—H2···Cl1	0.95	2.60	3.297 (5)	131
C3—H3···Cl1^i^	0.95	2.60	3.526 (5)	167
C5—H5···Se2	0.95	2.82	3.242 (5)	108
C5—H5···N2^ii^	0.95	2.45	3.179 (6)	134

Symmetry codes: (i) *x*, −*y*+1/2, *z*+1/2; (ii) *x*, −*y*+3/2, *z*+1/2.
