# Supplementary material for: Coupling between 2-pyridyl­selenyl chloride and phenyl­seleno­cyanate: synthesis, crystal structure and non-covalent inter­actions
Source: Acta Crystallogr E Crystallogr Commun. 2024 Sep 17;80(Pt 10):1024–8. doi: 10.1107/S2056989024008831 (PMC11451498; doi:10.1107/S2056989024008831)
Supplement: Supplementary file 3 [file e-80-01024-sup4.docx]

**Table S1**. Values of the density of all electrons – ρ(**r**), Laplacian of electron density – ∇^2^ρ(**r**) and appropriate λ_2_ eigenvalues, energy density – H_b_, potential energy density – V(**r**), Lagrangian kinetic energy – G(**r**), and electron localization function – ELF (a.u.) at the bond critical points (3, –1), corresponding to bifurcated chalcogen-hydrogen bonding Se···Cl^–^···H–C and chalcogen bonding Se···Se and Se···Cl^–^ in the obtained crystal structure, and estimated strength for these interactions E_int_ ≈ –V(**r**)/2 (kcal/mol).

| Contact^*^ | ρ(**r**) | ∇^2^ρ(**r**) | λ_2_ | H_b_ | V(**r**) | G(**r**) | ELF | E_int_ |
| --- | --- | --- | --- | --- | --- | --- | --- | --- |
| Se···Cl^–^ 2.933 Å | 0.025 | 0.058 | -0.025 | 0.000 | -0.014 | 0.014 | 0.159 | 4.4 |
| C–H···Cl^–^ 2.599 Å | 0.012 | 0.043 | -0.012 | 0.003 | -0.006 | 0.009 | 0.045 | 1.9 |
| Se···Se 3.943 Å | 0.005 | 0.013 | -0.005 | 0.000 | -0.002 | 0.002 | 0.025 | 0.6 |
| Se···Cl^–^ 3.380 Å | 0.011 | 0.030 | -0.011 | 0.001 | -0.005 | 0.006 | 0.062 | 1.6 |
| Se···Cl^–^ 3.229 Å | 0.014 | 0.037 | -0.014 | 0.001 | -0.007 | 0.008 | 0.074 | 2.2 |

^*^ The Bondi’s van der Waals radii for H, Se, and Cl atoms are 1.20, 1.90, and 1.75 Å, respectively^14^.
